# Medical and Physical Intelligence

**Published:** 1806-05

**Authors:** 


### MEDICAL AND PHYSICAL INTELLIGENCE.

Bishop MADISON, of America, has been enabled to ascertain that the Mammoth, or American Elephant, was an herbivorous animal.—In digging a well in Wythecounty, Virginia, after penetrating about five feet and a half from the surface, the labourers struck against the stomach of a Mammoth, the contents of which were in a state of perfect preservation, consisting of half masticated reeds, twigs, and grass, or leaves. The bishop adds, "there could be no deception; the substances were designated by obvious characters which could not be mistaken, and of which every one could judge; besides, the bones of the animal lay around, and added a silent, but sure confirmation." In addition to this fact it may be deserving of notice, that Mr. Francis Nevil, in his account of the elephantine teeth that were discovered in the north of Ireland, early in the eighteenth century, has mentioned some facts relative to the long preservation of vegetable matters, which seem worthy of our notice in this place: and the more so, as this gentleman's paper seems not to have excited any attention among the modern writers on the exuviæ of animals found in countries in which the living animals themselves are no longer seen. Some extravagant conjectures are mixed with Mr. Nevil's account: but these do not, in the least, invalidate the truth of what he says, relative to the bed upon which the Irish elephant was laid. "The place (says he) where this monster lay, was thus prepared, which makes me believe it had been buried, or that it had lain there since the deluge. It was about four feet under ground, with a little rising above the superficies of the earth, which was a plain under the foot of a hill, and about thirty yards from the brook, or thereabout. The bed whereon it lay had been laid with fern, with that sort of rushes here called sprits, and with bushes intermixed. Under this was a stiff blue clay, on which the teeth and bones were found: above this was, first, a mixture of yellow clay and sand, much of the same colour; under that a fine white sandy clay, which was next to the bed; the bed was for the most part a foot thick, and in some places thicker, with a moisture clear through it; it lay sad and close, and cut much like turf, and would divide into flakes, thicker or thinner as you would wish; and in every layer the seed of the rushes was as fresh as if new pulled, so that it was in the height of seed-time that those bones were laid there. The branches of the fern in every lay as we opened them, were very distinguishable, as were the seeds of the rushes and the tops of the boughs. The whole matter smelt very sour as it was dug, and tracing it I found it 34 feet long and about 20 or 22 feet broad."—"I forgot to mention that there was a great many nut-shells found about the bed, perhaps those might have been on the bushes which composed part of the bed."

Some members of the Galvanic Society at Paris, have ascertained that the galvanic action is augmented, 1. When the pile is exposed to a high temperature; 2. When it is plunged into flame, or in oxygen gas, or carbonic acid gas; 3. That the effects of the pile are not transmissible in vacuo, or that they are then scarcely perceptible, even by means of a condenser. It has been also ascertained that galvanism cannot be transmitted through smoke.

M. HERMAN, of Berlin, has examined the properties of different substances employed as Galvanic conductors, and has divided them into insulated bodies, into perfect and imperfect conductors, and into uni-polar and bi-polar bodies, according as their conductible property manifests itself at both, or only at one of the extremities of the pile.

Mr. HORNE has laid before the Royal Society of London, a paper describing a particular affection of the prostrate gland.—This disease, which occasioned so much pain, has hitherto been deemed irremediable; but it is now hoped, the cause having been discovered, that this physiological discovery may prove of incalculable advantage in relieving the sufferings of patients supposed to be labouring under the effects of calculi, and other urinary disorders.

On Wednesday the 16th of April, the Royal College of Surgeons adjudged the Jacksonian Prize for 1805, to John Hyslop, Esq. surgeon, Fenchurch-street, for the best Dissertation on "Injuries of the Head from external Violence."

Mr. BROOKES, in Blenheim Street, will commence his summer course of Lectures on Anatomy, Physiology, and Surgery, on Saturday, June 7, at two o'clock in the afternoon, and will be continued on Monday the 9th, and every subsequent morning at seven, Sunday excepted. The Dissecting Rooms will be opened at five in the morning.

Mr. CARPUE will commence his Lectures on Anatomy and Surgery early in June. Particulars may be known by applying to Mr. Carpue, No. 50, Dean Street, Soho.

Dr. HOOPER will commence a Summer Course of Lectures on the Theory and Practice of Physic, Materia Medica, and Pharmaceutical Chemistry, on Monday the 26th of May, at his Lecture Room, No. 26, Cork Street, Burlington Gardens, at half past eight o'clock in the morning. A Prospectus, with particulars, may be had at his house, No. 13, Holles Street, Cavendish Square.

Mr. JOHN TAUNTON, on the 24th of May, will commence the summer course of his Lectures on Anatomy, Physiology, Pathology, and Surgery.

Dr. REID will deliver the introductory Lecture to his Summer Course, on the Theory and Practice of Medicine, on Friday the 30th of May, at eight o'clock in the evening, at his house, Grenville-street, Brunswick-square; at which place the subsequent Lectures tures will also be delivered, at ten o'clock in the morning, every Monday, Wednesday, and Friday, until the conclusion of the Course.

At the beginning of this month will be published, a new edition (with some additions) of Dr. Clarke's Essays on the General Management of Pregnancy and Labor, and on the Inflammatory and Febrile Diseases of Lying-in Women, under the title of Essays on Subjects connected with the Practice of Midwifery, and the Diseases of Women and Children.

Dr. Willan has in the press a work on the Cow-pox, and on its varieties and anomalies, to be illustrated with a series of highly finished and accurate engravings, in the manner of his work on Cutaneous Diseases.

Dr. E. G. Clarke intends to publish, as soon as possible, Observations on the Diseases of the Army.

Mr. Hutchinson, of Southwell, is preparing for the press, An Essay on the Narcotic Powers of the Tartrite of Antimony introduced into the Syſtem by the cutaneous Absorbents. This Essay is expected to be publiſhed in July or August.

